# Inflammatory Proteins as Molecular Markers in the Diagnosis of Cervical Oncopathology

**DOI:** 10.17691/stm2021.13.4.07

**Published:** 2021-08-28

**Authors:** E.V. Kayukova, L.F. Sholokhov, A.M. Ziganshin, V.A. Mudrov

**Affiliations:** Associate Professor, Head of the Department of Oncology; Chita State Medical Academy, 39A Gorkogo St., Chita, 672000, Russia; Professor, Head of the Laboratory of Physiology and Pathology of the Endocrine System; Scientific Center for Family Health and Human Reproduction Problems, 16 Timiryazev St., Irkutsk, 664003, Russia; Associate Professor, Department of Obstetrics and Gynecology; Bashkir State Medical University, 3 Lenina St., Ufa, Republic of Bashkortostan, 450008, Russia; Associate Professor, Department of Obstetrics and Gynecology; Chita State Medical Academy, 39A Gorkogo St., Chita, 672000, Russia

**Keywords:** cervical cancer, cervical cancer diagnosis, SAA, ICAM-1, VCAM-1, sCD27

## Abstract

Imperfection of cytological diagnostics of cervical cancer has prompted the search for alternative methods of pathology detection.

**The aim of the study** was to evaluate the effectiveness of inflammatory proteins as molecular markers in the diagnosis of cervical oncopathology.

**Materials and Methods:**

A prospective controlled trial was conducted with three groups of women: group 1 (n=13) — with precancerous pathology (cervical intraepithelial neoplasia of grade III); group 2 (n=49) — patients with cervical cancer; group 3, control (n=13) — gynecologically healthy women (mean age — 30.0±4.4 years).

The material for the study was cervical epithelium, which was taken according to the standard technique using a cytobrush from the junction zone of cervical. The levels of inflammatory proteins (SAA, ICAM-1, VCAM-1, and sCD27) in the cervical epithelium were determined by flow cytometry.

**Results:**

Molecular criteria for the presence of precancerous pathology and cervical cancer have been found to be a 3.10 [1.31; 3.28] fold increase in SAA values (U=41.0, p=0.02), 2.62 [2.79 3.50] fold (U=137.0, p=0.001) in ICAM-1, 5.20 [3.84; 12.37] fold (U=138.5, p=0.001) in VCAM-1, and 4.32 [2.07; 5.02] fold (U=109.0, p<0.001) in sCD27 in cervical epithelium compared with the control group data. The COP (cervical oncoproblem) coefficient was developed to calculate the probability of cervical oncological pathology presence with the accuracy of 90%. An application for Android was created in Delphi development environment to simplify its calculation.

**Conclusion:**

The created technology makes it possible to establish the diagnosis in the shortest possible time and to optimize the treatment and diagnostic process by accelerating the examination period and improving its accuracy.

## Introduction

Cervical cancer (CC) is one of the most frequent tumors occurring in women worldwide. In Russia, this pathology accounted for 5.2% in the structure of oncological incidence, which corresponds to the third place after breast and skin cancers in 2018. The incidence of CC in Russia has increased by 23.3% over the last 10 years, while the mortality rate has increased by 2.84%. In 2019, the rate of active detection was 41.1%, taking into account tumors of visual localization; 33.4% of the patients were diagnosed with advanced forms of the disease [1, 2].

Presently in Russia, CC screening is carried out from the age of 21 years: in the age group of women 21–29 years — with cytological examination once in 3 years, at the age of 30–65 years — additionally with HPV test once in 5 years.

However, in recent years, an increasing number of critical publications on the effectiveness of cytology as a method of cervical screening and diagnosis of CC has appeared.

First of all, it concerns the organization of cervical screening, which is associated with low coverage of the population by the screening program. Second, there are imperfections in the method itself: large number of cytological classifications; low sensitivity (30–87%), which requires re-studies or clarifying diagnostics; errors in the technique of material sampling and interpretation of its results. Third, overdiagnosis of CC on the basis of HPV testing is observed, which is associated with the possibility of spontaneous virus elimination in the majority of infected persons [3, 4].

A technical improvement of the Pap test, such as liquid cytology, has not resulted in a meaningful improvement in the qualitative performance of cervical screening [4].

In some countries, computer-assisted screening techniques are used, which include hardware-assisted preparation of cytological material. Currently, ThinPrep (Hologic, Inc., USA) and SurePath (BD, USA) systems are known to be used in the diagnosis of cervical intraepithelial neoplasia, including high-grade ones. However, in the description of the results of studies on their use, the impact of these technologies on the rate of mortality from CC as the main indicator of the effectiveness of screening programs is not specified [5].

All of the above determines the relevance of the search for alternative methods of cervical screening and diagnosis of cervical pathology.

It is known that the main role in the pathogenesis of precancerous lesions and CC is played by the mechanisms of chronic virus-induced inflammation, in which some inflammatory proteins are actively involved:

SAA is an inflammation protein involved in both chronic inflammation and carcinogenesis through induction of metastasis cascade (potentiation of tumor adhesion and migration, stimulation of angiogenesis, activation of several ERK1/2, p38, and JNK mitotic signaling pathways) [6];

ICAM-1 and VCAM-1, intercellular and cellular adhesion molecules that regulate contacts between tumor cells, immune system cells, and vascular endothelium, are direct contributors to tumor progression [7];

sCD27 is a soluble form of CD27 transmembrane protein. Its interaction with CD70 ligand triggers antitumor immune response [8].

**The aim of this study** was to evaluate the effectiveness of the above inflammatory proteins as molecular markers in the diagnosis of cervical oncopathology.

## Materials and Methods

A prospective controlled study was conducted in which three groups of women were included: with pretumor pathology (cervical intraepithelial neoplasia grade III) — group 1 (n=13), age of patients — 32.0 [28.3; 36.5] years; with CC — group 2 (n=49), age of patients — 39.0 [35.6; 42.3] years; control — group 3 (n=13) of gynecologically healthy women at the age of 30.0 [25.5; 35.7] years.

The groups were randomized based on morphologically (for the comparison groups) and cytologically (for the control group) confirmed cervical epithelial status.

All subjects were informed about the conducted study and gave their written consent to participate in it. The study was carried out in compliance with the principles of the Declaration of Helsinki (2013) and approved by the Ethics Committee of Chita State Medical Academy (Russia).

The material for the study was cervical epithelium, which was taken according to the standard technique using a cytobrush from the junction zone of cervical epithelium.

By flow cytometry using Human Vascular Inflammation Panel 1-S/P (12-plex) with Filter Plate (Canada) and HU Immune Checkpoint Panel 1-S/P (10-plex) w/FP (Canada), levels of inflammatory proteins were determined in cervical epithelium: serum amyloid A (SAA), intercellular adhesion molecule 1 (ICAM-1), vascular cell adhesion molecule 1 (VCAM-1), and soluble CD27 (sCD27).

The statistical analysis of the study results was performed according to the principles of the International Committee of Medical Journal Editors (ICMJE) and the recommendations of Statistical Analysis and Methods in the Published Literature (SAMPL) [9, 10]. The analysis of normality of trait distribution, given the number of study groups equal to less than 50 women, was performed by estimating the Shapiro–Wilk criterion. Considering non-normal distribution of the signs in the variation series, statistical analysis was performed using non-parametric statistical methods: three study groups were compared by estimating the Kruskal–Wallis H-criterion, followed by pairwise comparison of statistically significant indicators using Mann–Whitney U-criterion. Considering Bonferroni correction, differences were considered statistically significant at p<0.017 [26]. The results of the study are presented as median, first and third quartiles — Me [Q1; Q3].

Binary logistic regression method was used to estimate the probability of cervical oncopathology presence. The diagnostic value of the developed model was determined by plotting the ROC curve with subsequent calculation of the area under it. Statistical processing of the study results was performed using an IBM SPSS Statistics v. 25.0 software package (USA).

## Results and Discussion

The following data were obtained in the study (Table 1).

**Table 1:** Levels of cervical epithelial proteins in the study groups (Me [Q1; Q3])

Cervical epithelial proteins	Groups			Statistical significance
	Control (n=13)	1^st^ (n=13)	2^nd^ (n=49)	
SAA (ng/ml)	1.0 [1.05; 1.71]	3.10 [2.24; 3.44]	2.18 [2.77; 3.50]	H=10.4 p=0.006
ICAM-1 (ng/ml)	1.07 [1.09; 1.77]	2.29 [2.04; 3.50]	2.80 [3.04; 3.81]	H=11.6 p=0.003
VCAM-1 (ng/ml)	0.15 [0.19; 0.38]	0.51 [0.94; 4.05]	0.78 [1.46; 2.35]	H=11.5 p=0.003
sCD27 (pg/ml)	8.13 [8.13; 14.17]	23.65 [29.62; 41.32]	35.09 [29.36; 40.83]	H=14.5 p=0.01

The SAA level in group 1 was 3.10 [1.31; 3.28] times (U=41.0, p=0.02) higher than the control index, but did not statistically significantly differ from the corresponding index in group 2 under study (U=256.0, p=0.78). Such results indicate that the SAA level is a nonspecific marker for the diagnosis of cervical malignant transformation; this can be explained by the participation of this protein both in the mechanisms of chronic inflammation and carcinogenesis.

The ICAM-1 value was maximal in group 2, which was 2.62 [2.79; 3.50] times higher than the corresponding value in the control group (U=137.0, p=0.001), but did not differ from values in group 1 (U=224.5, p=0.37).

The VCAM-1 level in group 2 was 5.20 [3.84; 12.37] times higher than in the control group (U=138.5, p=0.001), with no statistically significant differences with values in group 1 (U=248.5, p=0.67).

Our findings indicate active involvement of ICAM-1 and VCAM-1 molecules in cervical carcinogenesis.

The sCD27 amount was maximal in group 2 and exceeded its value in the control group by 4.32 [2.07; 5.02] times (U=109.0, p<0.001), but did not differ from that in group 1 (U=241.0, p=0.57). The detected increase in sCD27 amount in cervical epithelium of patients with precancerous diseases and CC indicates activation of cellular immunity; however, it may be assumed to be insufficient to avoid CC progression.

Based on binary logistic regression, an equation for the probability of cervical oncoproblem (COP) was generated:

$$COP=\frac{1}{1+e^{1\text{.2-0}.2\cdot \text{SAA-0}.02\cdot \text{ICAM-1-0}.5\cdot \text{VCAM-1-0}.1\cdot \text{SCD27,}}}$$

where 1.2 is the constant (regression coefficient *b*_0_); 0.2, 0.02, 0.5, and 0.1 are non-standardized *b* coefficients; *e* is the base of the natural logarithm (*e* ~2.72). A COP coefficient value of 0.7 or more was indicative of cervical oncopathology. A value of 0.5 or less corresponded to a healthy state of the cervical epithelium.

Based on the analysis of the logistic regression equation, the significance of the indicators in the structure of the diagnostic model was determined (Table 2).

**Table 2:** Significance of logistic regression indicators in the structure of the diagnostic model

Protein	B	Mean-square error	“Forest”	Degree of freedom	Statistical significance	Exp (B)
SAA	0.2	0.3	0.4	1	0.05	1.2
ICAM-1	0.02	0.4	0.04	1	0.009	1.2
VCAM-1	0.5	0.7	0.5	1	0.05	1.7
sCD27	0.1	0.5	4.4	1	0.004	1.1
Constant	–1.2	0.7	2.7	1	0.01	0.3

**Figure 1:**
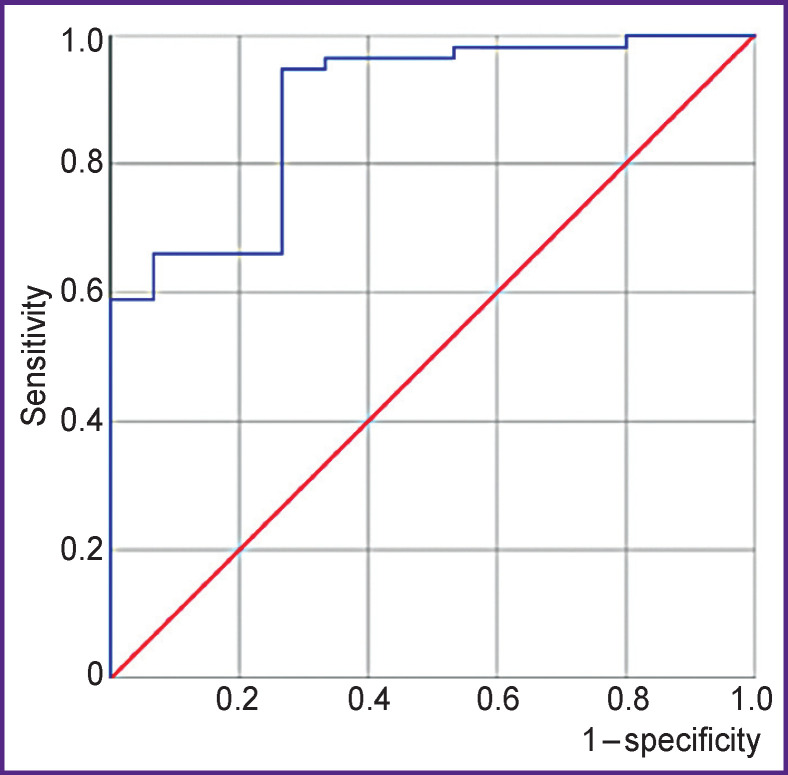
Area under the ROC-curve for the developed model

The sensitivity of the developed diagnostic model is 0.95, specificity — 0.73. The area under the ROC curve is 0.89 (95% CI 0.80–0.98); p<0.001. The standard error is 0.04 (Figure 1).

**Figure 2:**
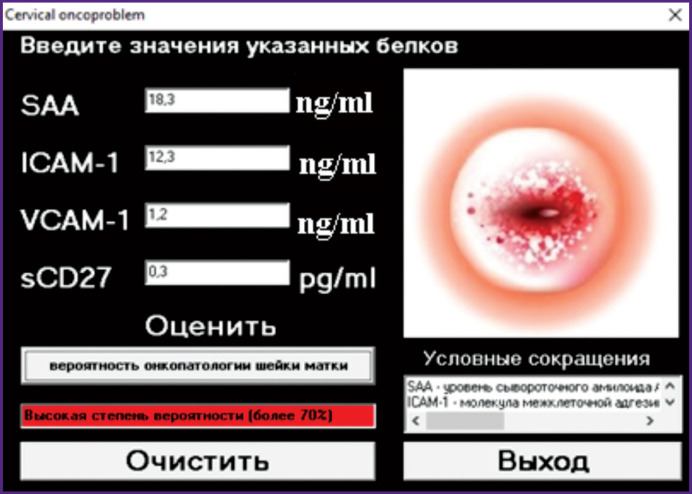
View of the window of the developed application

Given the complexity of the necessary calculations and to simplify the use of the proposed technology in everyday clinical practice, the application for Android in Delphi development environment (Delphi 10.3.3 Rio; Embarcadero, USA) was created. This application allows you to confirm the presence of cervical cancer in a particular patient in the shortest amount of time (Figure 2).

**Figure 3:**
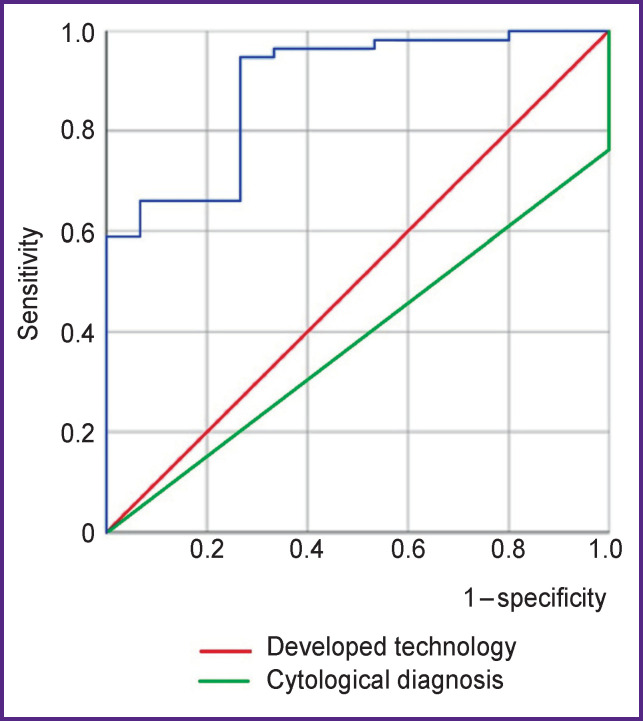
Area under the ROC-curve for the developed model and cytological diagnosis

Despite the fact that the functional relationship between marker levels and the presence of cervical cancer is not fully recognized, the resulting model predicts about 90% of true-positive cases. This figure is probably due to the persistence of inflammatory response in the area of malignant transformation in the cervical epithelium at the time of material sampling. Thus, the data obtained testify to the high informative value of the developed technique in comparison with the results of a traditional cytological examination (Figure 3).

Attention should be drawn to the fact that the area under the ROC curve for cytological examination is 0.38 (95% CI 0.24–0.53; p=0.2), which does not allow this method to be considered statistically significant.

**Figure 4:**
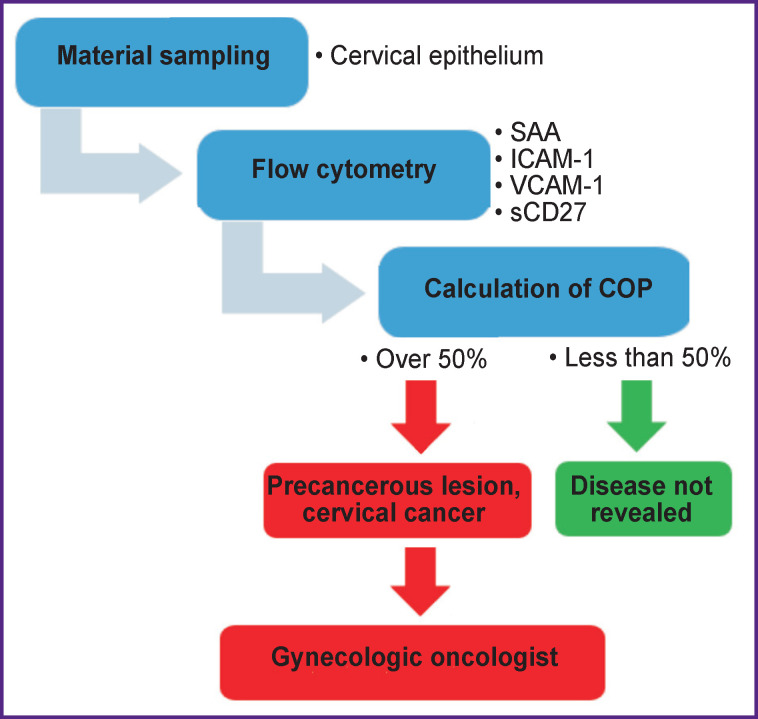
Algorithm scheme for screening a patient for cervical cancer

Figure 4 presents an algorithm of diagnostic measures to determine the presence of CC.

## Conclusion

Molecular criteria for the presence of precancerous pathology and cervical cancer may include a 3.10 [1.31; 3.28] fold increase in SAA values (U=41.0, p=0.02), 2.62 [2.79; 3.50] fold (U=137.0, p=0.001) in ICAM-1, 5.20 [3.84; 12.37] fold (U=138.5, p=0.001) in VCAM-1 and 4.32 [2.07; 5.02] fold (U=109.0, p<0.001) in sCD27 in cervical epithelium compared to the control group data.

The developed COP coefficient makes it possible to calculate the probability of the cervical cancer pathology presence with an accuracy of up to 90%, which is more than 2.2 times higher than that of cytological diagnostics.

The use of the developed diagnostic algorithm will make it possible not only to detect cervical oncopathology in a timely manner but also to optimize the treatment and diagnostic process.
